# Effect of Decompressive Craniectomy on Perihematomal Edema in Patients with Intracerebral Hemorrhage

**DOI:** 10.1371/journal.pone.0149169

**Published:** 2016-02-12

**Authors:** Christian Fung, Michael Murek, Pascal P. Klinger-Gratz, Michael Fiechter, Werner J. Z’Graggen, Oliver P. Gautschi, Marwan El-Koussy, Jan Gralla, Karl Schaller, Martin Zbinden, Marcel Arnold, Urs Fischer, Heinrich P. Mattle, Andreas Raabe, Jürgen Beck

**Affiliations:** 1 Department of Neurosurgery, University Hospital Bern, Bern, Switzerland; 2 Institute for Diagnostic and Interventional Neuroradiology, University Hospital Bern, Bern, Switzerland; 3 Department of Neurosurgery, University Hospital Geneva, Geneva, Switzerland; 4 Department of Neurology, University Hospital Bern, Bern, Switzerland; Heinrich-Heine University, GERMANY

## Abstract

**Background:**

Perihematomal edema contributes to secondary brain injury in the course of intracerebral hemorrhage. The effect of decompressive surgery on perihematomal edema after intracerebral hemorrhage is unknown. This study analyzed the course of PHE in patients who were or were not treated with decompressive craniectomy.

**Methods:**

More than 100 computed tomography images from our published cohort of 25 patients were evaluated retrospectively at two university hospitals in Switzerland. Computed tomography scans covered the time from admission until day 100. Eleven patients were treated by decompressive craniectomy and 14 were treated conservatively. Absolute edema and hematoma volumes were assessed using 3-dimensional volumetric measurements. Relative edema volumes were calculated based on maximal hematoma volume.

**Results:**

Absolute perihematomal edema increased from 42.9 ml to 125.6 ml (192.8%) after 21 days in the decompressive craniectomy group, versus 50.4 ml to 67.2 ml (33.3%) in the control group (Δ at day 21 = 58.4 ml, p = 0.031). Peak edema developed on days 25 and 35 in patients with decompressive craniectomy and controls respectively, and it took about 60 days for the edema to decline to baseline in both groups. Eight patients (73%) in the decompressive craniectomy group and 6 patients (43%) in the control group had a good outcome (modified Rankin Scale score 0 to 4) at 6 months (P = 0.23).

**Conclusions:**

Decompressive craniectomy is associated with a significant increase in perihematomal edema compared to patients who have been treated conservatively. Perihematomal edema itself lasts about 60 days if it is not treated, but decompressive craniectomy ameliorates the mass effect exerted by the intracerebral hemorrhage plus the perihematomal edema, as reflected by the reduced midline shift.

## Introduction

Intracerebral hemorrhage (ICH) remains a severe disease with mortality rates within 30 days of up to 50% [1,2]. Besides the initial hematoma volume [1], secondary hematoma expansion [3–5], intraventricular hemorrhage [6,7] and mechanisms of secondary brain injury—e.g. the development of a perihematomal edema (PHE) [8–10]–are responsible for the high morbidity and mortality. PHE, being part of the secondary brain damage in ICH, develops partly due to thrombin-mediated endothelial cell damage and inflammation [11–15] and red blood cell lysis [16–19]. Therapies like hypothermia or continuous infusion of hypertonic saline aim to reduce PHE [20–22]. Aggressive lowering of the blood pressure also decreases absolute PHE growth, at least in patients with volumes of intracerebral hemorrhages of about 10cc [10]. Decompressive craniectomy is a neurosurgical procedure that relieves intracranial pressure; it has been applied in ischemic stroke, cerebral sinus venous thrombosis (CSVT), aneurysmal subarachnoid hemorrhage and traumatic brain injury [23–26]. Recently, large decompressive craniectomy (DC) without hematoma evacuation showed a trend towards reduced mortality as compared to matched controls [27], and may be a beneficial treatment in selected cases [28]. The effect of DC without hematoma evacuation on PHE is unknown. Due to current ongoing trials on DC in ICH [29] further analysis of the time course and effect of PHE development in these patients is relevant. We therefore investigated PHE development in our previously published cohort of patients being treated with DC without hematoma evacuation for ICH [27].

## Materials and Methods

This retrospective data analysis was approved by the local ethics committee (Kantonale Ethikkommission Bern). The research was conducted in accordance with the principles of the Declaration of Helsinki. Because this was an anonymized retrospective analysis, the ethics committee did not require patients' informed consent.

This study is a secondary analysis of a previously published cohort investigating DC without hematoma evacuation for ICH in patients being treated at the Departments of Neurosurgery of the Bern University Hospital and the Geneva University Hospital between November 2010 and January 2012 [27]. Demographic, clinical and outcome data were assessed as previously described [27]. The criteria to perform surgery were at least one of the following: Glasgow Coma Scale (GCS) score <15, National Institutes of Health Stroke Scale (NIHSS) score >12, clinical deterioration compared to the admission status, or oculomotor nerve dysfunction. For each patient the decision to proceed with DC remained at the discretion of the treating surgeon [27]. DC was performed according to a previously published protocol with a DC diameter of at least 150mm in accordance with the rapid closure technique [30–32]. Control patients treated within the same time period were matched with respect to age, GCS score, hematoma volume, midline shift, and signs of herniation [27]. Best medical treatment for both groups was provided according to American Heart Association/American Stroke Association (AHA/ASA) guidelines [33]. Serial imaging was done upon admission and about 6 hours after first imaging to assess hematoma expansion, then within 24 hours after surgery. Further imaging was done as clinically indicated, and all computed tomography (CT) scans within 100 days after the hemorrhage were included. Day 1 was defined as the date of admission to the neurosurgical unit. Outcome was assessed at the outpatient clinic at 6 months according to the modified Rankin Scale (mRS) and dichotomized, with good outcome defined as mRS 0–4 [27]. This outcome measure was selected according to the pooled analysis of 3 randomized trials in patients with malignant middle cerebral artery infarction and 1 trial in CSVT [23,25].

### Volumetric analyses

Analyses of the hematoma and PHE were done using version 4.2.2 of the 3DSlicer (https://www.slicer.org/) by two coauthors (PPG and MF) who were blinded for clinical data. PHE and the hematoma were outlined semiautomatically using the level tracing tool and the software produced a three-dimensional dataset. Threshold ranges for hematoma were 44 to 100 Hounsfield units, and 5 to 33 Hounsfield units for edema [20]. Absolute hematoma and edema volumes were assessed at each time point and relative edema was calculated [34]. Maximum hematoma volume at 24 hours after surgery was used to calculate relative edema volumes. In DC patients the volumes were corrected for brain expansion, and volume of each hemisphere was analyzed. For correction, a ratio of the non-operated divided by the operated hemispheric volume was multiplied by the edema volume. Corrected relative edema volumes were calculated accordingly.

### Statistics

Growth curve analysis with a mixed-effects model was used to analyze the effects of DC on PHE development [35]. The overall edema curves were modeled with third-order polynomials with fixed effects of treatment-group on the intercept (baseline volume) and linear time terms (edema growth), and random effects of patients on the intercept and linear time terms to model individual differences in baseline volume and edema growth. The control group was treated as baseline and parameters were estimated for the DC group. Differences between treatment groups on days 1, 8, 21, and 28 were calculated by shifting the y-axis to the corresponding day and assessing parameter-specific p-values on the intercept using Satterthwaite’s approximation. All analyses were performed with RStudio (2013) and the “R language for statistical computing” using the lme4 package (version 1.0–5) [36]. P-values were calculated using t-test, Fisher-test, F-test and log-likelihood-ratio-test as appropriate.

## Results

Of the 27 patients included into the surgical study [27], 25 were included for edema analysis. One patient of each group was excluded because they received magnetic resonance imaging. The DC group comprised 11 patients (4 women, median age 48 [interquartile range (IQR) 33–59]), and the control group comprised 14 patients (7 women, median age 55.5 [IQR 47.8–69.5]). Despite exclusion of these two patients the two groups did not show a significant difference with respect to the matched parameters (Table 1). The etiology of the ICH is displayed in Table 1. One hundred twelve CT scans were analyzed.

**Table 1:** Demographic, clinical and radiological data of the two groups.

	DC group	Controls	P-value
Number (Men/Women)	11 (7/4)	14 (7/7)	
Median age in years, median (IQR)	48 (33–59)	56 (48–70)	P = 0.167
Etiology of intracerebral hemorrhage, (n)	Spontaneous (6)	Spontaneous (10)	
	Cerebral sinus venous thrombosis (1)	Cerebral sinus venous thrombosis (1)	
	Stroke (1)	Tumor (2)	
	Arteriovenous malformation (1)	Infection (1)	
	Dural AV fistula (1)		
	Herpes encephalitis (1)		
Median Glasgow Coma Scale score (IQR)	7 (4–10)	8 (3–13)	P = 0.807
NIHSS, median (IQR)	21 (20–27)	19 (12–31)	P = 0.419
Preoperative herniation, n	4	3	P = 0.43
Midline shift (mm) preoperative, median (IQR)	9 (5.7–10)	7.4 (0.8–12.6)	P = 0.557
Postoperative, median (IQR)	1 (0–4.8)		
Time from ictus to surgery (h), median (IQR)	15 (4–69)		

AV, atrioventricular; DC, decompressive craniectomy; IQR, interquartile range; n, number of patients; NIHSS, National Institutes of Health Stroke Scale.

Median volumes of ICH at admission and within 24 hours after surgery, respectively, were 59.6 ml (IQR 26.51–79.02) and 69.6 ml (IQR 27.62–79.02) for the DC group (means of 58.7 ml and 61.6 ml), and 36.4 ml (IQR 19.38–68.85, p = 0.70) and 47.6 ml (IQR 19.83–100.88, p = 0.78) for the control group (means of 52.0 ml and 60.3 ml).

### Perihematomal edema volume

**Fig 1:**
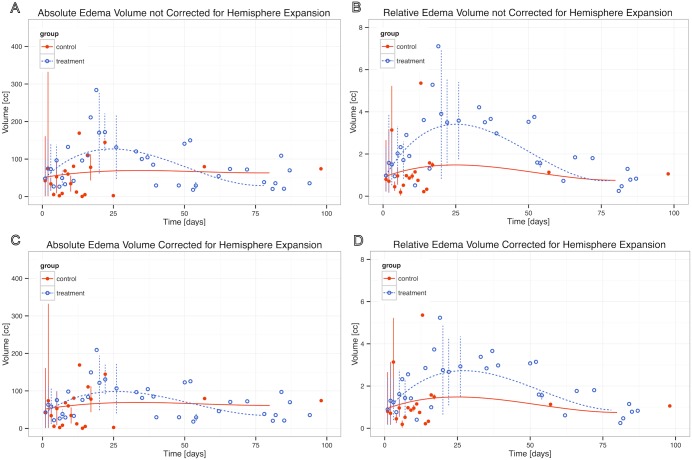
Absolute (A) and relative (B) perihematomal edema for decompressive craniotomy treatment and control groups, and corrected absolute (C) and corrected relative (D) perihematomal edema for the treatment and control groups.

Mean edema volume at admission was 42.9 ml in the DC group and 50.4 ml in the controls (Table 2, S1 Table). Throughout the first 3 weeks absolute PHE showed a larger increase in the DC group than in the control group, which was not statistically significant at day 8 (p = 0.149) but was statistically significant at day 14 (p = 0.047) and day 21 (p = 0.031) (Fig 1A, S2 Table).

**Table 2:** Absolute and relative edema volumes and corrected volumes.

		Day 1	Day 8	Day 14	Day 21
Absolute edema volume	Control	50.4	59.2 (17.5%)	63.7 (26.4%)	67.2 (33.3%)
	DC	42.9	91.7 (113.8%)	113.6 (164.8%)	125.6 (192.8%)
	Difference	7.5	32.5	49.9	58.4
	p-value	0.753	0.149	0.047	0.031
Relative edema volume	Control	0.89	1.23 (38.2%)	1.38 (98.0%)	1.47 (65.2%)
	DC	0.77	2.28 (196.1%)	2.96 (284.4%)	3.35 (335.1%)
	Difference	0.12	1.05	1.58	1.88
	p-value	0.798	0.025	0.003	0.001
Absolute edema volume corrected	DC	37.7	71.6 (89.9%)	87.3 (133%)	96.7 (156.5%)
	Difference	-12.7	12.4	23.6	29.5
	p-value	0.565	0.541	0.302	0.236
Relative edema volume corrected	DC	0.73	1.81 (147.9%)	2.32 (217.8%)	2.64 (261.6%)
	Difference	-0.16	0.58	0.94	1.17
	p-value	0.701	0.159	0.047	0.026

DC, decompressive craniectomy.

Mean absolute and relative edema volumes of the DC and control groups at different time points are shown in the upper rows. In the lower rows of the table the corrected mean absolute and relative edema volumes for the DC group are displayed.

Relative PHE on day 1 showed no significant difference between the DC and control groups (p = 0.80). However, statistically significant differences between the groups were found for day 8 (p = 0.025), day 14 (p = 0.003) and day 21 (p = 0.001). Peak absolute PHE edema volume reached 127 ml in the DC group on day 25 and 69.5 ml in the control group on day 35 (Fig 1B).

### Corrected perihematomal edema volumes

After DC the operated hemisphere had a median volume of 669 ml (IQR 616–726) compared to 513 ml (IQR 491–524) for the contralateral hemisphere.

Development of corrected absolute PHE volume (Fig 1C) is shown in Table 2. Changes in corrected absolute PHE volumes at post-operative dates were not significantly different between groups. Corrected relative edema volumes were significantly greater in the DC group at day 14 and day 21 (see Table 2). After correcting for hemispheric expansion, peak absolute PHE volume was 98.5 ml on day 26 (Fig 1D).

### Outcome

Eight patients (73%) in the DC group and 6 patients (43%) in the control group had a good outcome (P = 0.23).

## Discussion

Edema formation increased over time in all patients with ICH. The increase in PHE was significantly greater in DC patients than controls. Even after correcting for brain expansion the increased PHE in DC patients persisted for several weeks and was greater than expected. Previous MRI investigations and animal experiments supported a vasogenic origin of PHE in ICH rather than cytotoxicity or ischemia [37–40]. Due to the fact that hematoma evacuation was not performed in either group, an additional mechanism must be responsible for the difference in edema formation [41]. The difference in PHE formation might be due to the DC itself. One explanation might be a reduced intracranial pressure in DC patients as reflected by the significant reduction of postoperative midline shift from 9 mm to 1 mm [27]. Reduction of intracranial pressure and an increase in volume of the hemisphere on the side of the DC might lead to enlargement of the extracellular space with consequent reduction of interstitial fluid pressure, leading to increased bulk flow with increased edema formation [42]. Similar results were shown in a non-human model of experimental cerebral edema [43]. Cooper et al. also described increasing edema after DC in dogs, and proposed that the difference between the intravascular and interstitial pressure is the driving force [43]. Other mechanisms like clot retraction (early phase), activation of coagulation cascade and thrombin formation (first 2 days) and red blood cell lysis (after 3 days) should occur in both groups.

Attempts have been made to treat PHE. Wagner et al. showed that hypertonic saline significantly reduced absolute edema and mortality after ICH [22]; Kollmar et al. [20] and Staykov et al. [21] investigated the effect of hypothermia on edema and outcome, and both therapies showed less PHE development in the treatment arm. In contrast, DC seems to promote edema development, possibly without a negative effect on clinical outcome. This may result because decompression reduces herniation, which outweighs edema development. Although we did not evaluate the pre- and postoperative intracranial pressure (ICP) and therefore cannot describe the precise course of the ICP, we evaluated the pre- and postoperative midline shift. In our DC patients the midline shift returned from 9 mm to 1 mm after DC, indicating good decompressive effect with consequent lower intracranial pressure despite larger edema volumes [27].

Whether consideration and correction of hemispheric expansion after DC to calculate edema volume (absolute and relative) is necessary, is not clear. By correcting the edema volume for hemispheric expansion we wanted to rule out the possibility that larger edema volumes in DC patients occur solely due to expansion of the brain. After correction for hemispheric expansion the difference between the two groups was reduced, yet PHE in DC patients reached larger values, showing that the increased edema is not only a result of brain expansion.

In our opinion this limited patient number shows a PHE development that is significantly larger in DC patients compared to controls. This finding is reported for the first time. Due to the fact that the hematoma was not evacuated in either group, the previously mentioned mechanisms of PHE development should occur in both groups. Without proof, in DC patients an increased bulk flow might be responsible for increased edema development, yet further mechanisms have to be evaluated. DC itself might be a promoting factor for PHE development. In our opinion, the possibility of larger edema formation in the DC group should, for the moment, have no effect on the indication for DC in ICH. The results of the currently running randomized trials of DC for ICH (SWITCH and CHINA Studies) may shed more light on edema formation [29].

### Study limitations

The limitations of the study are its retrospective design and the involvement of only two centers. Also, the small sample size limits the power, and few CT scans were available beyond day 21. Another limitation is the distribution of CT scans, which were not performed at defined time points due to the retrospective nature of this analysis. In addition, the origin of ICH is heterogeneous, which may influence edema development. In addition to edema development due to the hemorrhage alone, CSVT, arteriovenous malformations and dural AV fistulas might cause an aggravation of edema by venous congestion/hypertension; a cytotoxic component can also augment edema formation in tumors and infections.

## Conclusion

Development of PHE is aggravated in ICH patients treated by DC and lasts for about 60 days. DC is associated with a significant increase in PHE compared to patients who have been treated conservatively. PHE itself is not treated but DC ameliorates the mass effect exerted by the ICH plus the PHE, as reflected by the reduced midline shift.

## Supporting Information

S1 Table The table shows the results of the semi-automatic measurement of the perihematomal edema and hematoma volume. The patients are numbered (1–25) and separated into the treatment and control groups (column B). Results of the perihematomal edema volume are marked as "type 2" and results of the hematoma volume as "type 3" (column C). The acquisitions are marked in the top row. Only days on which a CT scan has been made are listed in this table.
(XLSX)

S2 Table This table displays results of the statistical analysis done with "RStudio". Besides the absolute edema and perihematomal edema volumes, it also displays the calculation of the relative perihematomal edema volumes.
(HTML)
