# Supplementary material for: Effect of Decompressive Craniectomy on Perihematomal Edema in Patients with Intracerebral Hemorrhage
Source: PLoS One. 2016 Feb 12;11(2):e0149169. doi: 10.1371/journal.pone.0149169 (PMC4752325; doi:10.1371/journal.pone.0149169)
Supplement: S2 Table — Besides the absolute edema and perihematomal edema volumes, it also displays the calculation of the relative perihematomal edema volumes. (HTML) [file pone.0149169.s002.html]

Statistics | ICH-Edema


# Statistics | ICH-Edema

- 1 Results for initial hematoma-volume:
- 2 Absolute Edema Volume
  - 2.1 Not corrected for hemisphere expansion
    - 2.1.1 Random effects model equation
    - 2.1.2 Plot
    - 2.1.3 Results
  - 2.2 Corrected for hemisphere expansion
    - 2.2.1 Random effects model equation
    - 2.2.2 Plot
    - 2.2.3 Results
- 3 Relative Edema Volume
  - 3.1 Not corrected for hemisphere expansion
    - 3.1.1 Random effects model equation
    - 3.1.2 Plot
    - 3.1.3 Results
  - 3.2 Corrected for hemisphere expansion
    - 3.2.1 Random effects model equation
    - 3.2.2 Plot
    - 3.2.3 Results
- 4 Writeup

---

# 1 Results for initial hematoma-volume:

Control-Group  
- Mean: 51.9952  
- Median: 36.4026  
- IQR: 49.4701 (19.37-68.85)

Treatment-Group  
- Mean: 58.7171  
- Median: 59.6316  
- IQR: 52.5089 (26.51-79.02)

p = 0.7071

---


---

# 2 Absolute Edema Volume

## 2.1 Not corrected for hemisphere expansion

### 2.1.1 Random effects model equation

Volume\_Control = 50.3946 + 1.3035x + -0.0274x2 + 1.6397 × 10^-4^x3  
Volume\_Treatment = 42.8545 + 7.6769x + -0.2065x2 + 0.0014x3

### 2.1.2 Plot

### 2.1.3 Results

#### 2.1.3.1 Difference to baseline Model

```
Data: df
Models:
m.base: volume ~ (day + I(day^2) + I(day^3)) + (1 + day | patient)
m.intercept: volume ~ (day + I(day^2) + I(day^3)) + group + (1 + day | patient)
m: volume ~ (day + I(day^2) + I(day^3)) * group + (1 + day | patient)
            Df  AIC  BIC logLik deviance Chisq Chi Df Pr(>Chisq)
m.base       8 1232 1254   -608     1216                        
m.intercept  9 1234 1258   -608     1216  0.55      1       0.46
m           12 1234 1267   -605     1210  5.97      3       0.11
```

#### 2.1.3.2 Group Differences on specific days

|  | vol\_contr | vol\_treat | vol\_diff | p value |
| --- | --- | --- | --- | --- |
| Day 1 | 50.39465 | 42.85447 | -7.540182 | 0.7531119 |
| Day 8 | 59.15290 | 91.74596 | 32.593062 | 0.1490670 |
| Day 14 | 63.72237 | 113.57493 | 49.852561 | 0.0469871 |
| Day 21 | 67.20095 | 125.56137 | 58.360426 | 0.0312844 |

#### 2.1.3.3 Peak-Time and Volume

|  | Day | Volume |
| --- | --- | --- |
| control | 34.40 | 69.48 |
| treatment | 24.51 | 126.93 |

## 2.2 Corrected for hemisphere expansion

### 2.2.1 Random effects model equation

Volume\_Control = 50.3238 + 1.3174x + -0.0281x2 + 1.6762 × 10^-4^x3  
Volume\_Treatment = 37.6991 + 5.2658x + -0.135x2 + 8.588 × 10^-4^x3

### 2.2.2 Plot

### 2.2.3 Results

#### 2.2.3.1 Difference to baseline Model

```
Data: df
Models:
m.base: vol_corr ~ (day + I(day^2) + I(day^3)) + (1 + day | patient)
m.intercept: vol_corr ~ (day + I(day^2) + I(day^3)) + group + (1 + day | patient)
m: vol_corr ~ (day + I(day^2) + I(day^3)) * group + (1 + day | patient)
            Df  AIC  BIC logLik deviance Chisq Chi Df Pr(>Chisq)
m.base       8 1212 1234   -598     1196                        
m.intercept  9 1214 1239   -598     1196  0.00      1       0.95
m           12 1217 1250   -597     1193  2.81      3       0.42
```

#### 2.2.3.2 Group Differences on specific days

|  | vol\_contr | vol\_treat | vol\_diff | p value |
| --- | --- | --- | --- | --- |
| Day 1 | 50.32376 | 37.69905 | -12.62470 | 0.5648732 |
| Day 8 | 59.14769 | 71.62749 | 12.47980 | 0.5414679 |
| Day 14 | 63.71165 | 87.32414 | 23.61249 | 0.3023031 |
| Day 21 | 67.13140 | 96.71593 | 29.58453 | 0.2356423 |

#### 2.2.3.3 Peak-Time and Volume

|  | Day | Volume |
| --- | --- | --- |
| control | 33.34 | 69.18 |
| treatment | 25.92 | 98.47 |

---

# 3 Relative Edema Volume

The relative edema volume was based on the maximal hematoma volume.

## 3.1 Not corrected for hemisphere expansion

### 3.1.1 Random effects model equation

Volume\_Control = 0.8933 + 0.0525x + -0.0014x2 + 8.7356 × 10^-6^x3  
Volume\_Treatment = 0.7712 + 0.2356x + -0.0062x2 + 4.0961 × 10^-5^x3

### 3.1.2 Plot

### 3.1.3 Results

#### 3.1.3.1 Difference to baseline Model

```
Data: df
Models:
m.base: rel_edema ~ (day + I(day^2) + I(day^3)) + (1 + day | patient)
m.intercept: rel_edema ~ (day + I(day^2) + I(day^3)) + group + (1 + day | 
m.intercept:     patient)
m: rel_edema ~ (day + I(day^2) + I(day^3)) * group + (1 + day | 
m:     patient)
            Df AIC BIC logLik deviance Chisq Chi Df Pr(>Chisq)  
m.base       8 322 343   -153      306                          
m.intercept  9 322 346   -152      304  2.05      1      0.152  
m           12 317 349   -147      293 10.53      3      0.015 *
---
Signif. codes:  0 '***' 0.001 '**' 0.01 '*' 0.05 '.' 0.1 ' ' 1
```

#### 3.1.3.2 Group Differences on specific days

|  | vol\_contr | vol\_treat | vol\_diff | p value |
| --- | --- | --- | --- | --- |
| Day 1 | 0.8932641 | 0.7711578 | -0.1221064 | 0.7983038 |
| Day 8 | 1.2293418 | 2.2785789 | 1.0492371 | 0.0254757 |
| Day 14 | 1.3819146 | 2.9624469 | 1.5805323 | 0.0032524 |
| Day 21 | 1.4688342 | 3.3546507 | 1.8858165 | 0.0014993 |

#### 3.1.3.3 Peak-Time and Volume

|  | Day | Volume |
| --- | --- | --- |
| control | 25.011 | 1.481 |
| treatment | 25.225 | 3.413 |

## 3.2 Corrected for hemisphere expansion

### 3.2.1 Random effects model equation

Volume\_Control = 0.8919 + 0.0526x + -0.0014x2 + 8.7564 × 10^-6^x3  
Volume\_Treatment = 0.7284 + 0.1657x + -0.0041x2 + 2.5374 × 10^-5^x3

### 3.2.2 Plot

### 3.2.3 Results

#### 3.2.3.1 Difference to baseline Model

```
Data: df
Models:
m.base: rel_edema_corr ~ (day + I(day^2) + I(day^3)) + (1 + day | patient)
m.intercept: rel_edema_corr ~ (day + I(day^2) + I(day^3)) + group + (1 + day | 
m.intercept:     patient)
m: rel_edema_corr ~ (day + I(day^2) + I(day^3)) * group + (1 + day | 
m:     patient)
            Df AIC BIC logLik deviance Chisq Chi Df Pr(>Chisq)
m.base       8 294 315   -139      278                        
m.intercept  9 295 319   -138      277  0.65      1       0.42
m           12 295 327   -136      271  5.68      3       0.13
```

#### 3.2.3.2 Group Differences on specific days

|  | vol\_contr | vol\_treat | vol\_diff | p value |
| --- | --- | --- | --- | --- |
| Day 1 | 0.8919368 | 0.7283739 | -0.1635629 | 0.7005871 |
| Day 8 | 1.2291761 | 1.8054415 | 0.5762654 | 0.1589331 |
| Day 14 | 1.3823245 | 2.3173407 | 0.9350162 | 0.0473550 |
| Day 21 | 1.4696350 | 2.6424899 | 1.1728549 | 0.0257728 |

#### 3.2.3.3 Peak-Time and Volume

|  | Day | Volume |
| --- | --- | --- |
| control | 25.022 | 1.482 |
| treatment | 27.194 | 2.726 |

---

# 4 Writeup
